# Rheumatoid Arthritis‐Associated Interstitial Pneumonia Refractory to Initial Therapy: Successful Control Through Combined Anti‐Inflammatory, Antifibrotic and JAK‐STAT‐Targeted Treatment

**DOI:** 10.1002/rcr2.70378

**Published:** 2025-11-02

**Authors:** Takao Kodera, Yumiko Oka, Yuko Shirota, Motoki Kubota, Kei Soeda, Junichi Kameoka, Tomonori Ishii

**Affiliations:** ^1^ Division of Hematology and Rheumatology Tohoku Medical and Pharmaceutical University Sendai Miyagi Japan

**Keywords:** Baricitinib, interstitial lung disease, Nintedanib, rheumatoid arthritis, transforming growth factor beta

## Abstract

Connective tissue disease (CTD)–associated interstitial lung disease (ILD) accounts for a significant proportion of ILD cases, with rheumatoid arthritis (RA) being one of the most common underlying disorders. Although immunosuppressive therapy plays a central role in CTD‐ILD, its efficacy is limited in fibrotic ILDs, particularly those with a usual interstitial pneumonia (UIP) pattern. We report a case of RA‐associated ILD in an elderly woman who experienced acute disease progression despite ongoing treatment. A multimodal approach combining corticosteroids, tacrolimus, antifibrotic therapy (nintedanib) and the Janus kinase (JAK) inhibitor baricitinib led to marked clinical, radiological and biomarker improvement. This case underscores the potential benefit of a multi‐target strategy addressing both inflammation and fibrosis and suggests a possible role for JAK inhibition in refractory RA‐ILD.

## Graphical abstract

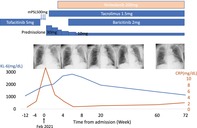

## 1 Introduction

Connective tissue disease (CTD)–associated interstitial lung disease (ILD) accounts for a significant proportion of ILD cases, with rheumatoid arthritis (RA) being one of the most common underlying disorders. RA‐ILD, particularly with a usual interstitial pneumonia (UIP) pattern, has been shown to carry a poor prognosis, reported 5‐year survival rate of only 20%–30% [1]. Nintedanib has demonstrated efficacy in slowing disease advancement of progressive fibrosing ILD, including RA‐ILD, but many patients have progressive disease. Here, we describe an elderly woman with RA‐ILD who showed insufficient response to corticosteroids, tacrolimus and nintedanib, but achieved marked improvement with the addition of the Janus kinase (JAK) inhibitor baricitinib. We also discuss potential molecular mechanisms underlying this therapeutic response, particularly the role of Transforming Growth Factor‐β (TGF‐β) as a pivotal mediator in the fibrotic process.

## 2 Case Report

**FIGURE 1:**
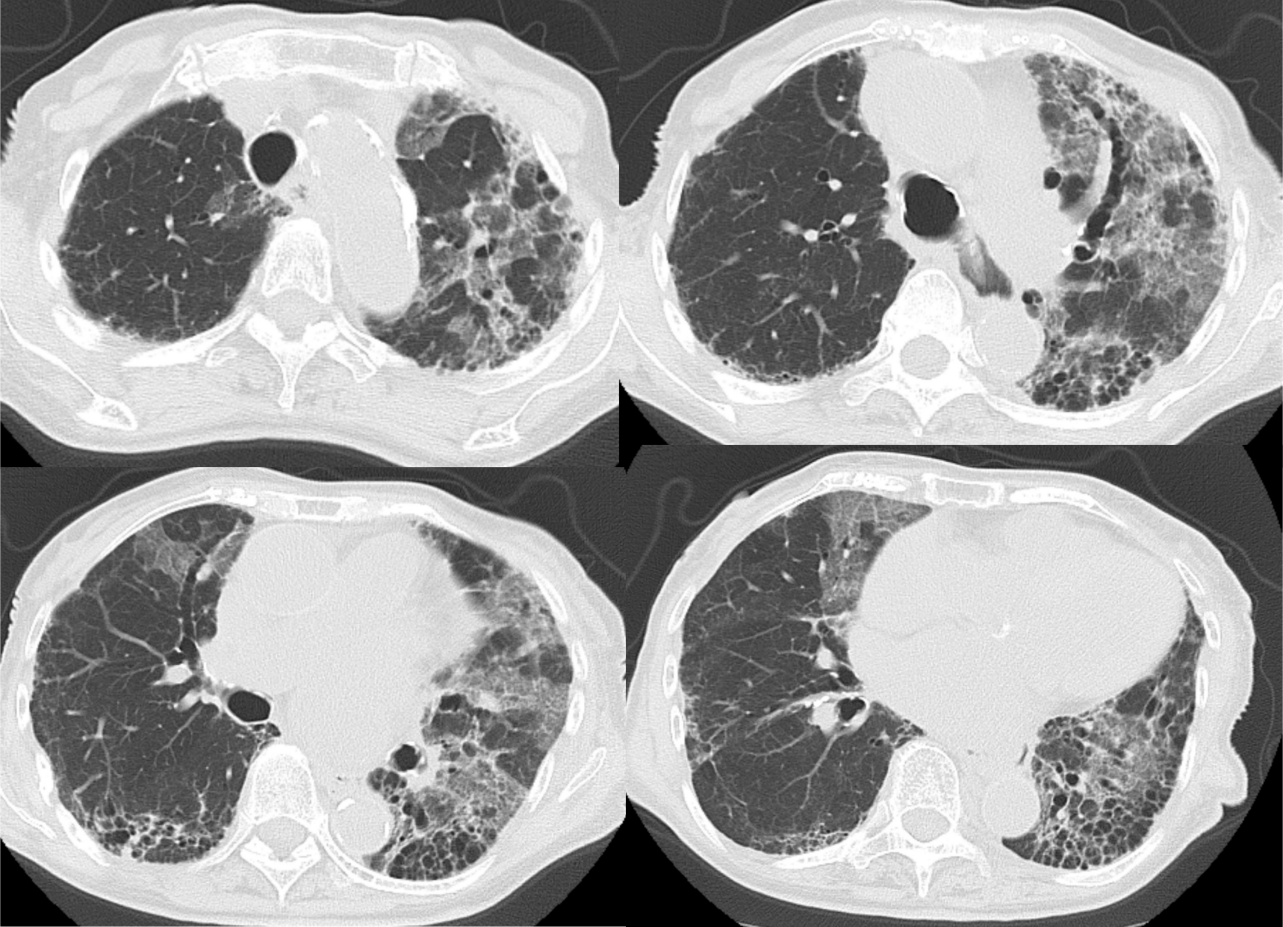
High‐resolution chest computed tomography (HRCT) image at the time of admission. The scan shows bilateral ground‐glass opacities and reticular changes predominantly in the lower lung fields, consistent with a progression of pre‐existing ILD.

A woman in her 80s with RA diagnosed at age 59 was initially treated with methotrexate (6 mg weekly) and sulfasalazine, but was referred to our institution due to suboptimal disease control. Adalimumab achieved a good clinical response; however, ILD had already developed. Due to concerns about drug‐induced pulmonary toxicity, treatment was deescalated to tacrolimus monotherapy in 2016. One year later, worsening arthritis prompted a switch to tofacitinib (5 mg daily), which achieved low disease activity. In February 2021, she developed progressive dyspnea and was admitted for the progression of ILD (Figure 1).

**FIGURE 2:**
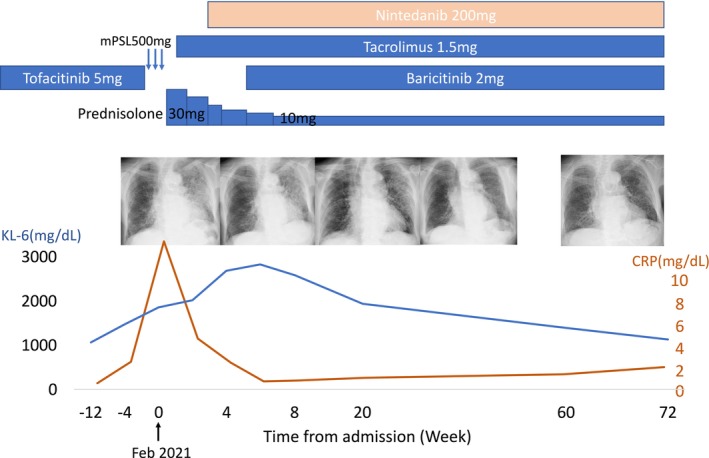
Clinical course of the patient, showing changes in serum KL‐6 and CRP levels over time in relation to treatment modifications. The graph illustrates the initiation and discontinuation of various medications, including baricitinib, tofacitinib, tacrolimus, nintedanib, methylprednisolone (mPSL) and prednisolone. Chest X‐ray findings are aligned with the timeline and demonstrate radiographic changes corresponding to disease activity and treatment response.

Serum KL‐6 (Krebs von den Lungen‐6), widely used in Japan as a biomarker of ILD, was approximately 1000 U/mL (cutoff 500 U/mL) prior to admission, but rose to 1856 U/mL at presentation. Tofacitinib was discontinued, and intravenous methylprednisolone (500 mg/day) was administered for 3 days, followed by tapering oral prednisolone (30 mg/day). Tacrolimus (1 mg/day) and nintedanib (200 mg/day) were added. Although disease progression was halted, oxygen therapy remained necessary and chest X‐ray showed no improvement. Despite ongoing treatment, KL‐6 continued to rise, peaking at 2830 U/mL. With positive CRP and joint pain, baricitinib (2 mg/day) was introduced, leading to rapid improvement in chest X‐ray and KL‐6, and discontinuation of oxygen therapy. Prednisolone was tapered to 10 mg/day, and the patient was discharged (Figure 2).

## 3 Discussion

We present a case of progressive RA‐ILD managed with therapies targeting a range of cellular mechanisms involved in inflammation and fibrosis. In the pathogenesis of ILD, profibrotic factors drive fibroblast migration, proliferation, activation and differentiation into myofibroblasts, leading to extracellular matrix (ECM) deposition and structural distortion of the lung. TGF‐β is a central mediator in this process, promoting both myofibroblast differentiation and ECM production. TGF‐β is produced by platelets, T lymphocytes, fibroblasts, monocytes and macrophages. Chronic stimulation by these inflammatory cells may contribute to persistent TGF‐β signalling and fibrosis. Therefore, suppressing the upstream inflammatory activity leading to TGF‐β production is considered important in managing RA‐ILD. Indeed, lower disease activity in RA has been shown to reduce ILD incidence [2].

TGF‐β signalling is closely interconnected with inflammatory cascades. Interleukin‐6 (IL‐6), a major pro‐inflammatory cytokine, activates JAK1 and JAK2 through its receptor, leading to phosphorylation and nuclear translocation of Signal Transducer and Activator of Transcription 3 (STAT3). STAT3 then directly or indirectly interacts with SMAD family member 3 (Smad3), the downstream mediator of TGF‐β signalling—a mechanism referred to as the STAT3‐Smad3 crosstalk [3]. At least part of the TGF‐β signalling cascade appears to depend on inflammatory STAT3 activation, implying that inhibition of STAT3 may also suppress TGF‐β–mediated fibrosis.

Given this interplay between inflammation and fibrosis, the therapeutic control of RA‐ILD may involve targeting the following four key pathways:

1. Suppression of inflammation (a source of TGF‐β) using corticosteroids or immunosuppressants.
2. Direct inhibition of TGF‐β.
3. Antifibrotic therapy (e.g., with nintedanib).
4. Inhibition of STAT3 activation to disrupt crosstalk between STAT3 and Smad3.

Baricitinib selectively inhibits JAK1 and JAK2, thereby attenuating IL‐6–mediated STAT3 activation. The patient had previously received tofacitinib, a JAK inhibitor with a similar mechanism, but disease control remained inadequate. In addition to steroid‐based suppression of inflammation, the combination of tacrolimus and nintedanib helped halt disease progression; however, neither imaging findings nor oxygenation capacity improved until baricitinib was initiated. This suggested that baricitinib provided further suppression of residual TGF‐β activity, leading to significant clinical and radiologic improvement.

A similar mechanism may explain the benefit observed in a clinical trial of nintedanib plus tocilizumab, an IL‐6 receptor antagonist, in systemic sclerosis‐associated ILD. In this study of 12 patients with progressive, treatment‐refractory ILD, HRCT showed disease stabilisation in all cases and marked improvement in two [4]. The effect was attributed to inhibition of IL‐6–mediated STAT3 activation and disruption of STAT3–Smad3 crosstalk, thereby attenuating TGF‐β signalling.

Of the four therapeutic targets proposed above, direct inhibition of TGF‐β remains the most challenging. Tacrolimus, in addition to its immunosuppressive effects, is expected to exert some inhibitory activity against TGF‐β; however, this effect is likely modest. TGF‐β knockout mice die shortly after birth, and clinical trials using anti‐TGF‐β antibodies in humans have been hindered by adverse effects such as stomatitis and valvular heart disease [5]. This reflects the essential role of TGF‐β in tissue repair and homeostasis, suggesting that precise modulation, rather than complete inhibition, is required.

Patients with CTD‐ILD present at varying stages of fibrosis, and the relevance of each pathogenic mechanism differs between individuals. Therapy should be tailored to the underlying disease, treatment response and presumed pathophysiology. Combination therapies with agents targeting TGF‐β signalling and fibrosis may offer effective strategies for refractory CTD‐ILD.

## Author Contributions

T.K. was the primary physician responsible for the patient's care, including treatment decisions, medication selection and clinical monitoring, and also drafted the manuscript. The other authors contributed to treatment planning, provided clinical input and advice, and were involved in patient care as supporting physicians. All authors reviewed and approved the final version of the manuscript.

## Ethics Statement

Ethical approval was obtained from the Research Ethics Committee for Life Science and Medical Research, Tohoku Medical and Pharmaceutical University (approval number: 2025‐4‐027‐0000).

## Consent

The patient described in this report is deceased. The authors declare that written informed consent for publication of this manuscript and accompanying images was obtained from the patient's daughter (next of kin), and that the signed consent form complies with the *Respirology Case Reports* requirements as outlined in the author guidelines.

## Conflicts of Interest

The authors declare no conflicts of interest.

## Data Availability

The data that support the findings of this study are available from the corresponding author upon reasonable request.
